# Patterns of multidrug resistant organism acquisition in an adult specialist burns service: a retrospective review

**DOI:** 10.1186/s13756-022-01123-w

**Published:** 2022-06-13

**Authors:** Heather Cleland, Lincoln M. Tracy, Alex Padiglione, Andrew J. Stewardson

**Affiliations:** 1 Victorian Adult Burns Service, Alfred Health, Melbourne, Australia; 2 School of Public Health and Preventive Medicine, Monash University, Melbourne, Australia; 3 Department of Infectious Diseases, Alfred Health, Melbourne, Australia; 4 Department of Infectious Diseases, The Alfred Hospital and Central Clinical School, Monash University, Melbourne, Australia

**Keywords:** Burn injury, Multi-drug resistant bacteria

## Abstract

**Background:**

Multidrug resistant organisms (MDROs) occur more commonly in burns patients than in other hospital patients and are an increasingly frequent cause of burn-related mortality. We examined the incidence, trends and risk factors for MDRO acquisition in a specialist burns service housed in an open general surgical ward, and general intensive care unit.

**Methods:**

We performed a retrospective study of adult patients admitted with an acute burn injury to our specialist statewide tertiary burns service between July 2014 and October 2020. We linked patient demographics, injury, treatment, and outcome details from our prospective burns service registry to microbiology and antimicrobial prescribing data. The outcome of interest was first MDRO detection, stratified into the following groups of interest: methicillin-resistant *Staphylococcus aureus* (MRSA), vancomycin-resistant Enterococcus (VRE), two groups of *Pseudomonas* (carbapenem resistant, and piperacillin-tazobactam or cefepime resistant), carbapenem-resistant *Acinetobacter* species, *Stenotrophomonas maltophilia*, carbapenem-resistant Enterobacteriaceae (CRE), and extended-spectrum beta-lactamase producing Enterobacteriaceae (ESBL-PE). We used a Cox proportional hazards model to evaluate the association between antibiotic exposure and MDRO acquisition.

**Results:**

There were 2,036 acute admissions, of which 230 (11.3%) had at least one MDRO isolated from clinical specimens, most frequently wound swabs. While acquisition rates of individual MDRO groups varied over the study period, acquisition rate of any MDRO was reasonably stable over time. Carbapenem-resistant *Pseudomonas* was acquired at the highest rate over the study period (3.5/1000 patient days). The 12.8% (29/226) of MDROs isolated within 48 h were predominantly MRSA and *Stenotrophomonas.* Median (IQR) time from admission to MDRO detection was 10.9 (5.6–20.5) days, ranging from 9.8 (2.7–24.2) for MRSA to 23.6 (15.7–36.0) for carbapenem-resistant *P. aeruginosa*. Patients with MDROs were older, had more extensive burns, longer length of stay, and were more likely to have operative burn management. We were unable to detect a relationship between antibiotic exposure and emergence of MDROs.

**Conclusions:**

MDROs are a common and consistent presence in our burns unit. The pattern of acquisition suggests various causes, including introduction from the community and nosocomial spread. More regular surveillance of incidence and targeted interventions may decrease their prevalence, and limit the development of invasive infection.

**Supplementary Information:**

The online version contains supplementary material available at 10.1186/s13756-022-01123-w.

## Introduction

Risk of death after burn injury has decreased in high income countries in recent decades, but infection remains a major cause of morbidity and is the major cause of in-hospital mortality [1]. In keeping with other health care settings and conditions, the emergence of antimicrobial resistance poses increasing challenges in the management of burns patients [2]. Bacteria with clinically important multidrug resistant phenotypes such as ﻿*Staphylococcus aureus* and various gram-negative infectious agents, in particular ﻿*Pseudomonas aeruginosa, Acinetobacter baumannii, Escherichia coli, Klebsiella pneumonia,* and *Enterobacter cloacae,* are common in burns services, which house patients with extensive skin loss and open wounds, decreased immune function, prolonged antibiotic use, invasive treatments, and long length of stay. These patient characteristics increase the risk of colonisation by, and infection with, multidrug resistant organisms (MDROs), and contribute to the poor outcomes associated with difficult to treat infections due to MDROs [3, 4]. MDROs occur more commonly in burns patients than in other hospital patients [5] and are an increasingly frequent cause of burn-related mortality [6].

A recent review of infection control measures to manage MDRO outbreaks in burns units, including removing patients and closing down the unit, showed that even the most comprehensive measures to eradicate MDROs may not be successful [7]. Thus, infection prevention and antibiotic stewardship initiatives designed to minimize the development and acquisition of MDROs are fundamental to best practice burns care. A systematic review of potentially modifiable risk factors for MDRO acquisition has identified antibiotic use, as well as hospital interventions more generally associated with increased risk of infection (urinary or intravascular catheters, mechanical ventilation, and hydrotherapy) as targets for prevention efforts. Strategies minimising the risk of MDRO acquisition in burns also include early wound excision and closure, meticulous wound management, and environmental control [4].

Other general aspects of infection prevention and control also have specific implications for burn care, including infrastructure design, models of care, isolation precautions, and cleaning regimens [8]. However, consensus on these issues is lacking, with the relative value of many basic practices, technologies, and design features in burns units undetermined [9, 10]. In contrast, the value of antibiotic stewardship in ensuring appropriate treatment of infection and managing de-escalation is well established, especially in combination with consistently applied infection control practices [11].

In order to ensure infection prevention and management efforts are well targeted and patients treated appropriately for clinical infection, it is necessary to have an understanding of patterns of infection and colonisation that are specific to individual settings. Additionally, the incidence and associations of acquisition of MDROs can act as indicators of quality of care and support quality improvement initiatives. In order to better understand the occurrence of bacterial MDROs and potential strategies for their prevention and management in our specialist statewide tertiary referral burns service, we aimed to examine incidence, trends, and risk factors for MDRO acquisition. We also examined the impact of antibiotic use and timing on MDRO acquisition.

## Methods

### Study setting and population

The Victorian Adult Burns Service (VABS) is a specialist adult burns service providing the statewide service for adult patients (≥ 16 years) in the Australian state of Victoria. The population of Victoria was 6,462,019 in 2017 [12]. Victoria has a regionalised, hierarchical trauma system, which ensures transfer of patients with severe burns to the specialist service. Previous research has shown that 98% of adult patients with severe burn injury are managed at the VABS [13]. In addition, many patients with less severe burns are cared for in this service. The VABS manages patients who require critical care in a general open intensive care unit (ICU), and ward patients are housed in an open general surgical ward that also accommodates plastic surgery patients. The service has a policy of routine surveillance swabbing of wounds on admission and at dressing changes at least weekly until healed or patient discharged. All adult patients admitted with an acute burn injury to the VABS between July 2014 and October 2020 and entered into the VABS database were included in this study.

### Data sources and data management

Admission, demographic (age and gender), injury event (cause and intent), injury severity (i.e., the percentage of total body surface area [%TBSA] burned), management, and in-hospital outcome (discharge disposition and hospital length of stay [LOS]) data were extracted from the VABS database. This database routinely captures epidemiological, quality of care, treatment and outcome data for all patients admitted to the service.

The %TBSA burned was reported as a continuous variable (i.e., 0–100) and categorised into two groups: 0–19.9%, and ≥ 20% TBSA, with the latter group defined as having a major burn injury. The primary cause of burn injury was dichotomoised to identify patients who sustained a flame burn, the most common cause of burn injury in adult patients in Australia and New Zealand. Injury intent was dichotomised to identify patients who sustained an unintentional injury. Discharge disposition was dichotomised to identify patients who were discharged to another hospital or healthcare facility as an additional indicator of injury severity. Hospital LOS (reported in days) was calculated from date and time of admission and discharge.

The hospital microbiology database was searched for specific organisms isolated from these patients during their inpatient stay. Data on the timing of the swab, where the specimen was collected (i.e., in theatre, on the ward, etc.), specimen type, and the organism(s) identified in the specimen were extracted. The MDRO groups of interest were: methicillin-resistant *Staphylococcus aureus* (MRSA), vancomycin-resistant Enterococcus (VRE), two groups of *Pseudomonas* (carbapenem resistant [Group 1] and piperacillin-tazobactam or cefepime resistant [Group 2]), carbapenem-resistant *Acinetobacter* species, *Stenotrophomonas maltophilia*, carbapenem-resistant Enterobacteriaceae (CRE), and extended spectrum beta lactamase producing Enterobacteriaceae (ESPL-PE). Rectal screening swabs were excluded. Specimens were grouped based on the location from which they were collected: wound, respiratory (including sputum and bronchoalveolar lavage), blood (including catheter tip cultures), or urine. Only the first isolate of each species of an organism was recorded. The number of unique MDRO organisms and organism groups for each patient was calculated. Time to isolation was calculated from date and time of admission and specimen collection data. The time to isolation was reported as a continuous variable (in days) and was also dichotomosed according to whether the specimen was isolated within 48 h of admission.

Antibiotic exposure data was available for the sub-group of patients admitted between October 2018 and October 2020. Their hospital electronic medical records were searched for non-topical antibacterial drugs. The name and date of first administration for each antibiotic was extracted from the hospital’s electronic prescribing record system. Antibiotic administration was examined in all patients for whom data were available. Further analysis of antibiotic exposure in patients who had MDROs isolated was also conducted. Exposure to antibiotics which were active against most or all isolates of an organism other than the resistant phenotype of interest (dubbed ‘Standard’ antibiotics), was determined for patients with each of the MDRO groups of interest (Additional file 2: Table S1). Time to first exposure for each unique antibiotic was calculated using date and time of admission and order data.

### Statistical analysis

Data from the VABS, microbiology, and pharmacy databases were linked using patient name, birth date, and medical record number. Summary statistics were used to describe the profile of patients who did and did not develop an MDRO. Frequencies and percentages were used for categorical variables, while mean and standard deviation or median and interquartile range (IQR) were used for continuous variables depending on the skewness of the data. Differences between patients who did and did not develop an MDRO were assessed using chi-squared or Mann Whitney *U* tests, as appropriate. A *p*-value < 0.05 was considered statistically significant. The number of MDRO containing specimens was calculated for each MDRO group of interest and overall and reported using frequencies. The rate of MDRO acquisition per 1000 bed days and 95% confidence intervals (CIs) were calculated for the overall sample and for each MDRO group of interest individually. The association between antibiotic exposure and MDRO acquisition was evaluated using a Cox proportional hazards model, where antibiotic exposure was considered as a time-dependent covariant. The resulting hazard ratio (HR) and 95% CI was reported. Data handling and statistical analysis was performed using Stata Version 14.0 (StataCorp, College Station, Texas, USA) and in the R statistical environment version 4.0.3 [14]. Figures were produced in Excel 2016 (Microsoft, Redmond, Washington, USA) and in the R statistical environment version 4.0.3 [14] using the *tidyverse*, [15] *ggdist* [16], *gghalves* [17], *survival* [18, 19], and *survminer* packages [20].

### Ethics approval

The Alfred Human Research Ethics Committee granted ethics approval for this study (Project Number 154/20).

## Results

There were 2,036 acute admissions to the unit between July 2014 and October 2020, 230 (11.3%) of whom had at least one MDRO isolated from a clinical specimen. Of these, 160 acquired one MDRO, 43 acquired two MDROs, and 17 acquired three MDROs; the remaining patients acquired four or more MDROs. Patients with MDROs were older with more extensive burns. Patients with a major burn injury accounted for 10.1% of the total patient population, but 38.6% of patients with an MDRO. There was a positive relationship between length of hospital stay and MDRO identification. A greater proportion of patients with an MDRO underwent a burn wound management procedure in the operating theatre, while a smaller proportion of patients with an MDRO were discharged to home (Table 1).

**Table 1:** Patient characteristics

	All patients (n = 2036)	No MDRO (n = 1806)	Any MDRO (n = 230)	*p*-value
Age, median (IQR) years	41 (28, 57)	40 (27, 56)	49 (33, 65)	< 0.001
Male	1497 (73.5%)	1338 (74.1%)	159 (69.1%)	0.11
TBSA, median (IQR) %	4.0 (2.0, 10.0)	4.0 (1.5, 8.0)	14.0 (6.0, 30.0)	< 0.001
Major burn injury	203 (10.1%)	115 (6.4%)	88 (38.6%)	< 0.001
Flame burn	1213 (59.6%)	1060 (58.7%)	153 (66.5%)	0.023
Unintentional injury	1889 (93.1%)	1685 (93.7%)	204 (88.7%)	0.005
Procedure in theatre	1430 (70.5%)	1208 (67.1%)	222 (97.8%)	< 0.001
Discharged to other hospital	890 (43.7%)	715 (39.6%)	175 (76.1%)	< 0.001
LOS, median (IQR) days	7.4 (3.1, 13.7)	6.5 (2.8, 11.5)	27.9 (14.9, 51.1)	< 0.001

Data presented as frequency (percentage) unless otherwise specified

*IQR* interquartile range; *MDRO* multi-drug resistant organism; *LOS* length of stay; *TBSA* total body surface area

*p*-value relates to comparisons between patients who did and did not develop an MDRO

MDROs were most frequently isolated from wound swabs. There were 323 wound swabs which were positive for an MDRO. MDROs were isolated from 13 blood cultures, 21 respiratory samples, and 12 urine specimens (Table 2).

**Table 2:** Number of unique MDRO specimens by organism and specimen type

Organism group	Wound	Respiratory	Blood	Urine
MRSA	67	6	0	0
VRE	27	< 5	< 5	0
*Pseudomonas* (Group 1)	83	8	< 5	< 5
*Pseudomonas* (Group 2)	15	< 5	< 5	< 5
CR *Acinetobacter* species	14	0	< 5	0
*Stenotrophomonas maltophilia*	65	< 5	< 5	< 5
CRE	9	0	0	< 5
ESBL-PE	43	< 5	0	< 5
Total	323	21	13	12

*CR* Carbapenem-resistant; *CRE* Carbapenem-resistant Enterobacteriaceae; *ESBL-PE* Extended spectrum beta lactamase producing Enterobacteriaceae; *MDRO* multi-drug resistant organism; *MRSA* Methicillin-resistant *Staphylococcus aureus*; *VRE* Vancomycin-resistant Enterococcus; *Pseudomonas aeruginosa* groups are defined by resistance to carbapenems (Group 1) or either piperacillin-tazobactam or cefepime (Group 2)

Some characteristics of patients with MDROs varied by the specific organism group they acquired. However, increasing size of burn, a wound management procedure in the operating theatre, discharge to another hospital (typically for rehabilitation) and increasing LOS were associated with all MDROs.

**Fig. 1:**
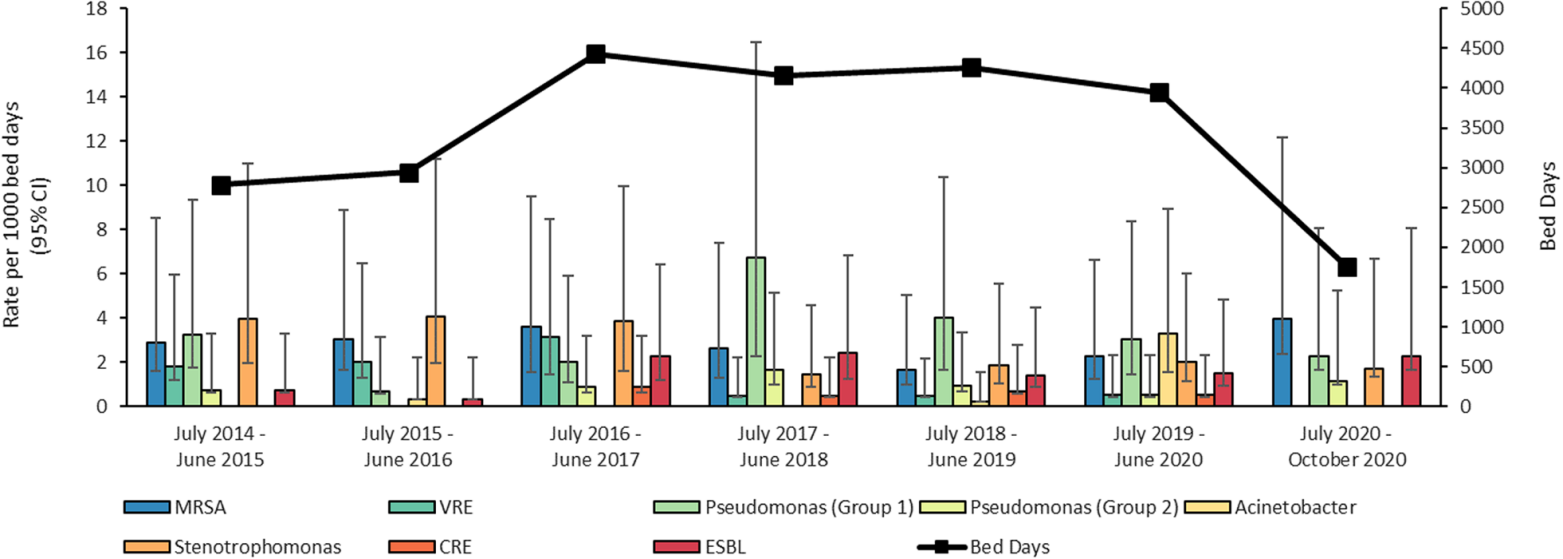
Rate of MDRO acquisition per 1000 bed days by MDRO type. Error bars represent 95% confidence intervals. *CRE* Carbapenem-resistant Enterobacteriaceae; *ESBL* Extended spectrum beta lactamase; *MDRO* multi-drug resistant organism; *MRSA* Methicillin-resistant *Staphylococcus aureus*; *VRE* Vancomycin-resistant Enterococcu*s*. *Pseudomonas aeruginosa* groups are defined by resistance to carbapenems (Group 1) or either piperacillin-tazobactam or cefepime (Group 2)

Rates per 1000 patient days for each organism by year varied (Fig. 1). In the 2017/18 year, the rate (95% CI) of carbapenem-resistant *Pseudomonas* was 6.7 (4.5–9.7)/1000 patient days. In the 2016/17 period, the VRE rate was 3.2 (1.7–5.3)/1000 patient days: in subsequent years this decreased to 0.5/1000 days. In 2019–2020, carbapenem-resistant *Acinetobacter* species, previously an uncommon occurrence in the burns unit, had a rate of 3.3 (1.8–5.7)/1000 days (13 cases), when preceding and subsequent years had zero or one case. In this study, *Acinetobacter* species isolates were all *A. baumannii*, except for one, which was *A. haemolyticus*, from a blood culture*.*

The total rates of MDROs showed no change over time (Additional file 1: Fig. S1). The MDRO with the highest rate over the study period was carbapenem-resistant *Pseudomonas*, at 3.5 (2.8–4.3)/1000 patient days (Additional file 3: Table S2).

**Fig. 2:**
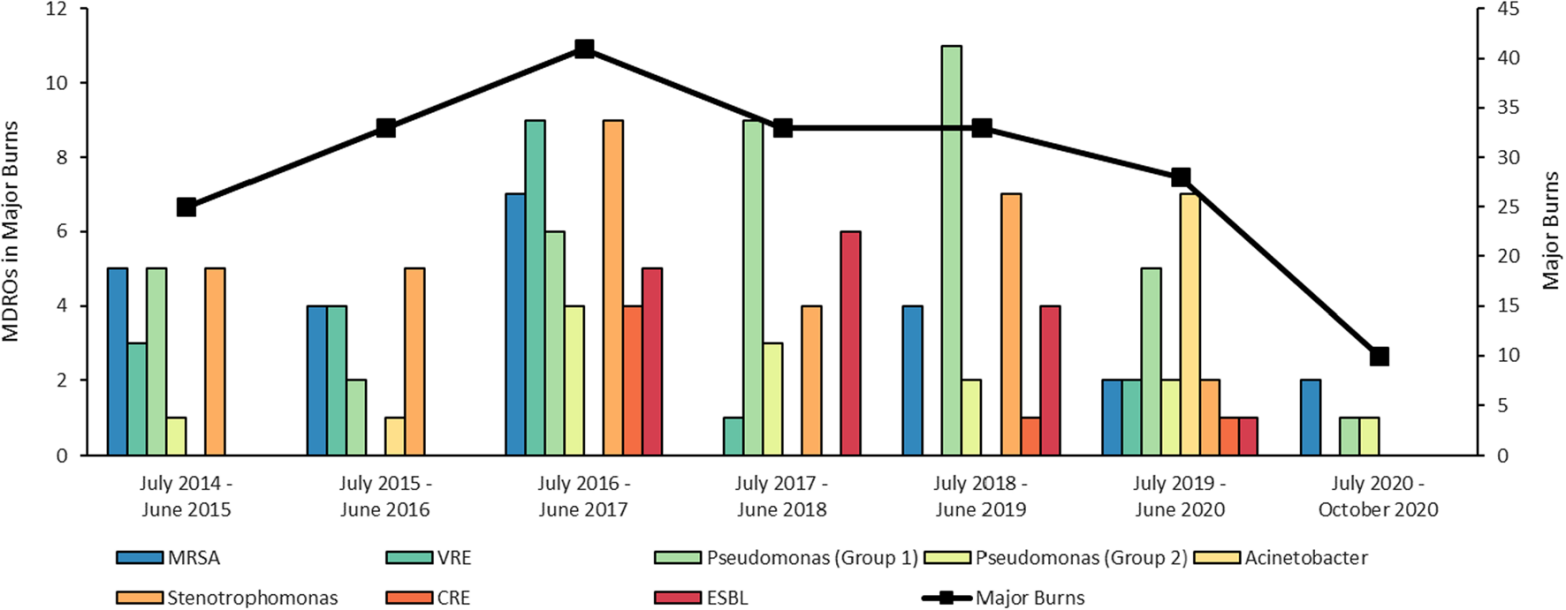
MDROs in major burn patients by MDRO type. *CRE* Carbapenem-resistant Enterobacteriaceae; *ESBL* Extended spectrum beta lactamase; *MDRO* multi-drug resistant organism; *MRSA* Methicillin-resistant *Staphylococcus aureus*; *VRE* Vancomycin-resistant Enterococcus. *Pseudomonas aeruginosa* groups are defined by resistance to carbapenems (Group 1) or either piperacillin-tazobactam or cefepime (Group 2)

Figure 2 shows the number of MDROs per year by type in patients with burns ≥ 20% TBSA. There were 203 patients with major burns admitted over the study period, an average of 2.7 patients per month. Resistant *P. aeruginosa* and *S. maltophilia* were isolated in every year of the study in patients with major burns, however other organisms (*Acinetobacter* species, CRE, ESBLs) were less consistently isolated. MRSA and VRE were absent from this group of patients for one 12 month period each.

### Time to isolation of MDRO

**Fig. 3:**
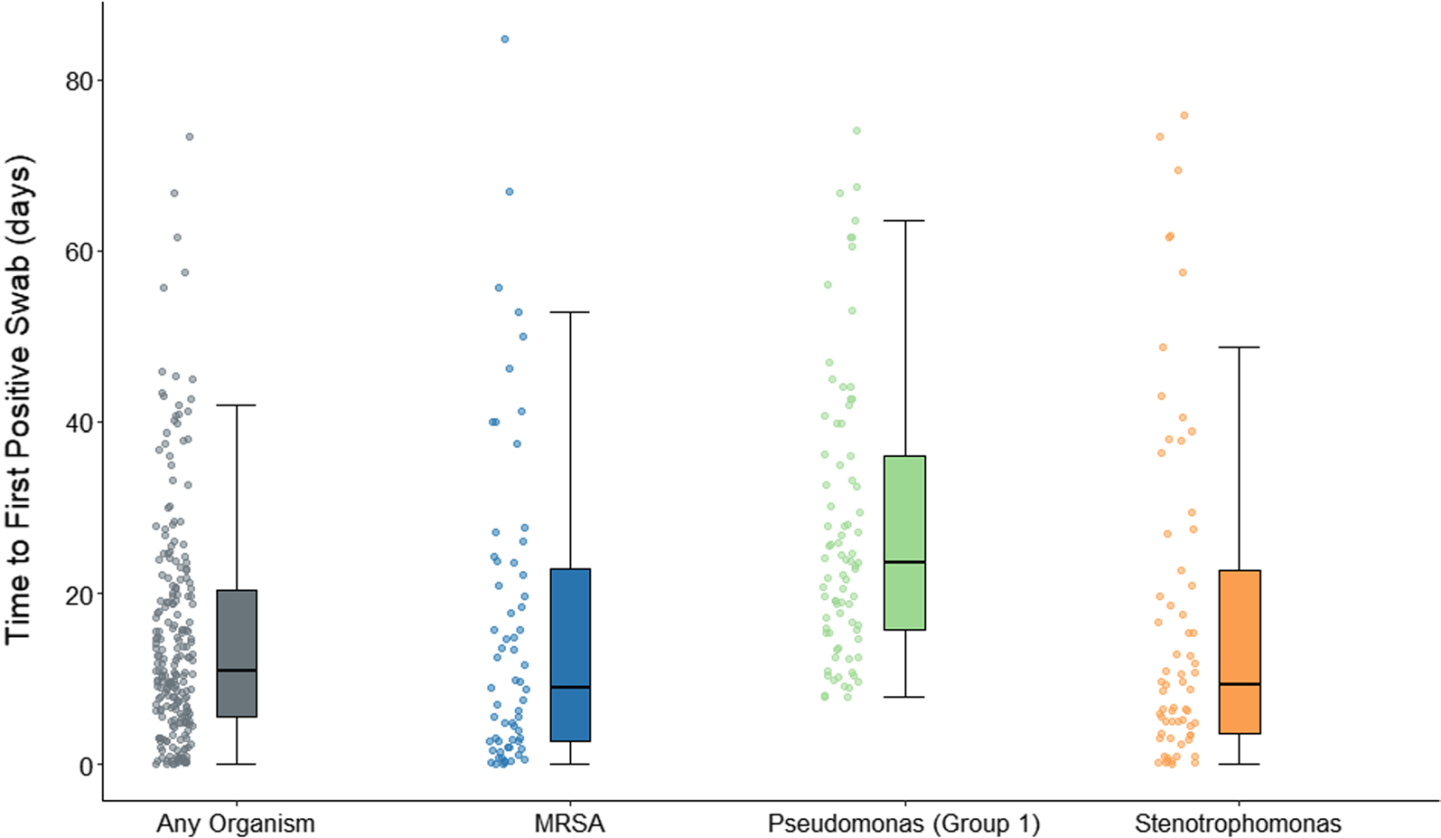
Time to first MDRO isolation (positive swab) since admission. *MDRO* multi-drug resistant organism; *MRSA* Methicillin-resistant *Staphylococcus aureus*. *Note* Patients could develop more than one MDRO during their admission. *Pseudomonas aeruginosa* (Group 1) is defined by resistance to carbapenems. *Note* Y-axis capped at 80 days; data beyond this value are not presented

Twenty-nine (12.8%) MDROs were isolated from specimens collected within 48 h of admission. These were predominantly MRSA (n = 15) and *Stenotrophomonas* (n = 10). No multi-resistant specimens of *P. aeruginosa*, *Acinetobacter* species or VRE were isolated within 48 h after admission (Additional file 4: Table S3). Median time to first positive clinical specimen varied according to organism type, with MRSA, ESBL-PEs and *Stenotrophomonas* less than 10 days, and carbapenem-resistant *P. aeruginosa* and CRE more than 3 weeks post admission (Fig. 3).

Antibiotic exposure data was available for 730 patients over a two-year period to October 2020. Ninety of these patients (12.3%) had an MDRO isolated from a clinical isolate. Three hundred and thirty-seven of the 730 (46.2%) patients received antibiotics. Twenty-five patients who did not have antibiotics had an MDRO isolated, 16 of whom had either a *P. aeruginosa* or *Acinetobacter* species. The relationship between developing an MDRO and exposure to antibiotics active against most isolates of an organism apart from the resistant organism of interest (Standard antibiotic) was examined. Of 81 instances of MRSA, *Pseudomonas*, or an ESBL-PE acquisition, 24 (30%) were not associated with prior exposure to Standard antibiotics (Table 3). There were insufficient numbers to analyse exposure to Standard antibiotics using Cox proportional hazards models. Exposure to any antibiotic was not associated with MDRO acquisition (HR [95% CI] = 1.35 [0.79, 2.28], *p* = 0.30).

**Table 3:** ‘Standard’ antibiotic use in patients developing MDRO, October 2018–October 2020

Organism	N	Antibiotic exposure† before MDRO isolation	Time in days between antibiotic exposure and MDRO isolation‡
MRSA	24	15 (62.5%)	23.7 (6.0–33.7)
VRE	< 5	< 5	–
Pseudomonas (Group 1)	33	24 (72.7%)	21.8 (14.2–35.5)
Pseudomonas (Group 2)	8	7 (87.5%)	17.0 (14.0–35.5)
CR *Acinetobacter* species	14	< 5	–
CRE	< 5	< 5	–
ESBL-PE	16	11 (68.8%)	7.8 (2.9–22.7)

Exposure before antibiotic data reported as frequency (percentage). Time between antibiotic exposure and MDRO isolation data reported as median (IQR). Excludes missing data

^†^Standard’ antibiotics refer to antibiotics that are active against most/all isolates of specific organism other than the resistant phenotype of interest

^‡^Excludes patients who developed MDRO before exposure to category 1 antibiotics

*Carbapenem-resistant CRE* Carbapenem-resistant Enterobacteriaceae; *ESBL-PE* Extended spectrum beta lactamase producing Enterobacteriaceae; *IQR* interquartile range; *MDRO* multi-drug resistant organism; *MRSA* Methicillin-resistant Staphylococcus aureus; *VRE* Vancomycin-resistant Enterococcus; *Pseudomonas aeruginosa* groups are defined by resistance to carbapenems (Group 1) or either piperacillin-tazobactam or cefepime (Group 2)

## Discussion

In this study, 11.3% of burns patients had an MDRO isolated from a clinical specimen during their admission. Comparisons with other units are difficult, due to different populations and whether or not the study focussed on infections only or included colonisation. Apart from excluding rectal swabs, our study made no attempt to distinguish the two. MDROs were most commonly isolated from wounds, with isolates from respiratory samples, blood, and urine being far less common. This largely reflects the wound surveillance swabbing policy in our unit, rather than relative incidence of wound infections. Van Lengeveld et al., in their single-centre review of adults and children admitted to their burn ICU, compared patients with infection due to MDROs with those with infections due to susceptible organisms. They found 47 of 1355 patients (3.5%) developed an MDRO infection. The commonest infectious complications in burns are urinary tract infections, pneumonia, wound infections, and bloodstream infections, although the relative frequency of these varies, potentially due to variable definitions of wound infection and different patient populations [3, 4, 21]. Factors associated with acquisition of MDROs in our unit are increasing age, size of burn, increasing LOS and operating theatre procedures, in keeping with other reports [3, 21–23].

### Incidence and time of isolation for different organisms

In our study, the commonest MDRO isolates were *P. aeruginosa*, followed by MRSA. Rates for different organisms varied considerably from 3.5/1000 bed-days for carbapenem-resistant *Pseudomonas* to 0.5 (0.2–0.9)/1000 bed-days for CRE. The commonest pathogens reported in burns units more generally are *A. baumannii, P. auruginosa, Klebsiella pneumonia, S. aureus,* and less commonly, *Enterococcus and Enterobacter* species [1]. In comparison to other studies, we recorded few *Acinetobacter* species [24–26]. A systematic review of risk factors for Gram negative MDROs reflects the clinical significance of *Acinetobacter* in burn patients, with seven of 11 studies focussed on *Acinetobacter* [22]. Following a previous outbreak of infection and colonization with gram‐negative pathogens carrying a metallo‐β‐lactamase gene in our hospital, prescription of meropenem is restricted [27]. Acinetobacter has consistently been a less common organism, however carbapenem resistant *P. aeruginosa* comprises the largest single group of MDROs in our service.

We also presented the rate of *S. maltophilia* isolation*,* which is rarely reported on in the burns literature. *S. maltophilia* was one of the three commonest organisms isolated in our study: 15% were isolated in the first 48 h, possibly indicating environmental pre-hospital acquisition. Reports of *Stenotrophomonas* in burns patients are few, but a study from Taiwan reported 14 burns patients with *Stenotrophomas* bacteraemia and a higher incidence in burns patients than non-burns patients in their hospital. They reported four deaths in association with polymicrobial sepsis [28]. In our study, of 73 isolates, fewer than five isolates each were detected from respiratory samples or blood. Despite a reputation as an opportunistic pathogen, usually infecting immunocompromised hosts, some strains have the potential to develop enhanced virulence in humans, and clusters in hospital populations suggest a capacity for spread in healthcare settings [29, 30]. The frequent isolation of *Stenotrophomonas* in our patients is a cause for some concern, indicating as it does possible hospital transmission, and an association with prolonged antibiotic use, with the potential to cause invasive infection.

### Time to isolation

Bacterial colonisation and infection of burn wounds typically occurs early after injury and initially more commonly with gram-positive organisms. With increasing LOS, gram-negatives come to predominate in wounds and hospital treatment related infections, along with increasing antibacterial resistance patterns [4, 21]. The pattern of isolation of different species in our study reflects this usual pattern. The median time (IQR) from admission to isolation in our cohort was 10.9 (5.6–20.5) days: 12.8% of these were isolated within 48 h after admission, and are more likely to have been acquired in the community. In their review of healthcare associated infections after burn injuries, van Duin et al*.* reported a median time of 38 (17–77) days from admission to first MDRO isolation. Median time to first isolation of MRSA was 11.5 days (3–33), compared with 9.8 (2.7–24.2) in our cohort [21]. Although the bulk of acquisitions were identifed within the first three weeks after admission in our cohort, they occurred throughout the hospital stay. The wound swabbing policy has provided unique information which identifies the extent of MDRO colonisation of patients in our service. As most MDROs were isolated from wounds which were not systemically treated in the absence of clinical signs of infection, they potentially persist for prolonged periods, especially in patients with extensive injuries, resulting in a high prevalence rate, with the ongoing risk of nosocomial transmission.

### Antibiotic use

We examined the association between exposure to antibiotics which were active against a specific organism other than the MDRO phenotype of interest (Standard antibiotics). Our analysis did not show an association between these antibiotics and MDRO acquisition, possibly due to low numbers, nor did it show an association between any antibiotic exposure and MDRO isolation. This finding is in contrast with other studies: a recent systematic review of risk factors for acquisition of gram-negative MDROs showed an increased pooled odds ratio of 7.00 (2.77–17.67) associated with exposure to extended spectrum cephalosporins, and 6.65 (3.49–12.69) for carbapenem exposure [31]. Another study showed > 10% incidence of bowel colonisation with ESBL gram negative organisms in hospital in-patients exposed to cephalosporin monotherapy and concluded that antibiotic resistance is an inescapable effect of antibiotic therapy [32].

Non-lactose-fermenting gram-negative organisms such as *P. aeruginosa*, frequently possess intrinsic resistance mechanisms, including low membrane permeability, and multiple genetic resistance determinants. Resistant strains are selected for during antibiotic use through removal of ‘competing’ organisms and sensitive strains, but acquisition of resistance mechanisms, and nosocomial transmission are other ways patients acquire these organisms [33]. In our study, exposure to Standard antibiotics was not a prerequisite for development of an MDRO: carbapenem-resistant *Pseudomonas* was isolated in the absence of exposure to selective antibiotic pressure in 27% of isolates. It is possible that the lack of association of MDROs with prior antibiotic exposure in our cohort is due, at least in part, to acquisition driven by nosocomial spread rather than de novo generation of resistance.

### Significance

Our study of unique clinical specimens positive for MDROs in acute burns patients indicates a consistent presence of these organisms within the service. The burns ward is located on an open general surgical ward shared with other services, and has common wound management and bathing areas. Despite periods of increased prevalence, no ‘outbreaks’ have been declared, although increased cleaning and isolation protocols are put in place when highly resistant organisms are identified. In addition to consistent infection control protocols, active consistent antibiotic stewardship is particularly needed in burns units, given that antibiotic exposure is an established and potentially modifiable risk factor for MDRO acquisition in critically ill patients [31]. An antibiotic stewardship team is part of our burns unit and provides direction for antibiotic prescribing, especially in more complex cases. However recent unpublished data from our unit indicates a high level of prolonged peri-operative 1st generation cephalosporin prescriptions that lack specific indications, and requires more oversight; especially in view of a recent systematic review finding that evidence for peri-operative antibiotic prophylaxis is lacking in burns patients [34]. Additionally, dosing regimens in complex burns patients require expert determination to achieve adequate treatment levels and minimize the risk of developing bacterial drug resistance [35]. A recent study of ICU antibiotic stewardship in Toronto, Canada, indicated that burns ICU staff were both more likely to receive suggestions from the antibiotic stewardship team, and more likely to reject those suggestions [36]. Given the specific complexities of diagnosing and treating multiple infections in burns patients, this finding suggests the need for consistent and senior staffing in stewardship and burn teams to develop shared understanding of clinical issues and decision making in this patient group.

While broad principles may be applicable to many environments, specific infection control practices and antibiotic prescribing are commonly hospital and unit specific, dictated by patient population, model of care, infrastructure, and microbiological resistance profiles.

### Limitations

This study did not identify isolates associated with invasive wound infections, the incidence of which will be lower than positive swabs. However, the demonstration of risk factors for acquisition of MDROs and their prevalence in the unit provides information to target at-risk patients and a basis for measuring improvements in prevalence associated with infection control measures. No molecular typing was done to investigate possible transmission. Antibiotic prescription data was available for a small subset of patients, and limited analysis was undertaken.

## Conclusion

MDROs are a common and consistent presence in our burns unit. The pattern of acquisition suggests various causes, including introduction from the community and nosocomial spread. More regular surveillance of incidence and targeted interventions may decrease their prevalence, and limit the development of invasive infection. Current infrastructure does not support best infection control measures.

## Supplementary Information

**Additional file 1: Fig. S1.** Rate of MDRO acquisition per 1000 bed days by year. Error bars represent 95% confidence intervals. MDRO = multi-drug resistant organism.

**Additional file 2: Table S1.** Antibiotics considered to be active against most/all isolates of each organism (or group) other than the resistant phenotype of interest (‘Standard’ antibiotics).

**Additional file 3: Table S2.** Rate of MDRO Acquisition per 1000 Bed Days by organism, July 2014 – October 2020.

**Additional file 4: Table S3.** Time to First MDRO Isolation since Admission.

## Data Availability

The datasets generated and analysed during the current study are not publicly available due to the requirement to protect patient confidentiality.

## Abbreviations

MDRO: Multidrug resistant organism
MRSA: Staphylococcus *aureus*
VRE: Vancomycin-resistant Enterococcus
CRE: Carbapenem-resistant Enterobacteriaceae
ESBL-PE: Extended-spectrum beta-lactamase producing Enterobacteriaceae
VABS: Victorian adult burns service
ICU: Intensive care unit
%TBSA: Total body surface area
LOS: Length of stay
IQR: Interquartile range
CI: Confidence intervals
HR: Hazard ratio
