# Supplementary material for: Patterns of multidrug resistant organism acquisition in an adult specialist burns service: a retrospective review
Source: Antimicrob Resist Infect Control. 2022 Jun 13;11:82. doi: 10.1186/s13756-022-01123-w (PMC9195457; doi:10.1186/s13756-022-01123-w)
Supplement: Supplementary file 1 — Additional file 1: Fig. S1. Rate of MDRO acquisition per 1000 bed days by year. Error bars represent 95% confidence intervals. MDRO = multi-drug resistant organism. [file 13756_2022_1123_MOESM1_ESM.docx]

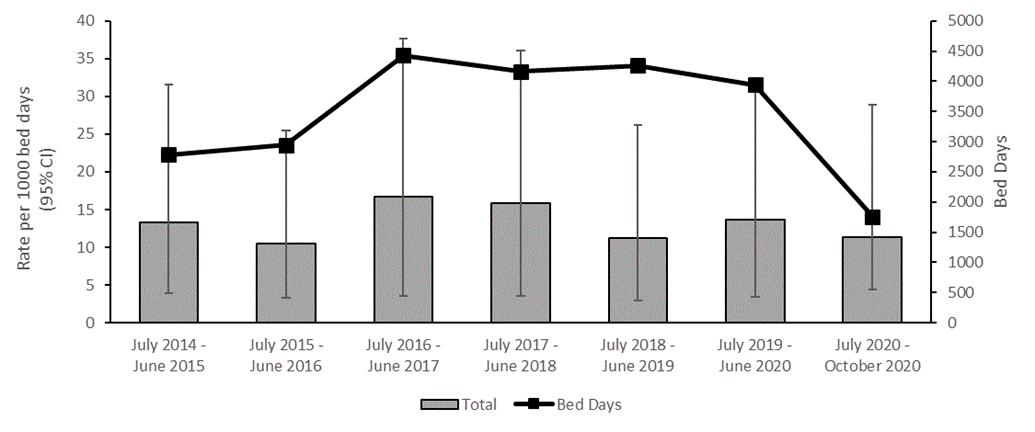


**Supplementary Figure 1.** Rate of MDRO acquisition per 1000 bed days. Error bars represent 95% confidence intervals. MDRO = multi-drug resistant organism.
