# Supplementary material for: Patterns of multidrug resistant organism acquisition in an adult specialist burns service: a retrospective review
Source: Antimicrob Resist Infect Control. 2022 Jun 13;11:82. doi: 10.1186/s13756-022-01123-w (PMC9195457; doi:10.1186/s13756-022-01123-w)
Supplement: Supplementary file 2 — Additional file 2: Table S1. Antibiotics considered to be active against most/all isolates of each organism (or group) other than the resistant phenotype of interest (‘Standard’ antibiotics). [file 13756_2022_1123_MOESM2_ESM.docx]

**Supplementary Table 1**: Antibiotics considered to be active against most/all isolates of each organism (or group) other than the resistant phenotype of interest (‘Standard’ antibiotics).

| **MRSA** | **VRE** | ***Pseudomonas auruginosa* (Group 1)** | ***Pseudomonas auruginosa* (Group 2)** | ***Acinetobacter* species** | ***Stenotrophomonas maltophilia*** | **CRE** | **ESBL-PE** |
| --- | --- | --- | --- | --- | --- | --- | --- |
| ertapenem  imipenem  meropenem  cefepime  cephalexin  cephalothin  cephazolin  flucloxacillin  amox/clav  pip/taz  moxifloxacin | vancomycin  teicoplanin  amoxicilin  ampicillin  benzylpenicillin  amox/clav  pip/taz  moxifloxacin  tigecycline | amikacin  gentamicin  tobramycin  imipenem  meropenem  cefepime  ceftazidime  ceftolozane  ceftolozane-tazobactam  aztreonam  pip/taz  ciprofloxacin | amikacin  gentamicin  tobramycin  imipenem  cefepime  ceftazidime  ceftolozane  aztreonam  pip/taz  ciprofloxacin | amikacin  imipenem  meropenem  cefepime  ceftolozane-tazobactam  minocycline | NA | amikacin  gentamicin  tobramycin  ertapenem  imipenem  meropenem  cefepime  ceftazidime  ceftolozane  ceftol/taz  ceftriaxone  cephalexin  nitrofurantoin  trim/sulf  amox/clav  pip/taz  ciprofloxacin  moxifloxacin  norfloxacin | cefepime  ceftazidime  ceftriaxone  cephalexin  cephalothin  cephazolin  aztreonam  nitrofurantoin  trim/sulf  amoxicilin  ampicillin  amox/clav  pip/taz  moxifloxacin  norfloxacin |
