# Supplementary material for: Patterns of multidrug resistant organism acquisition in an adult specialist burns service: a retrospective review
Source: Antimicrob Resist Infect Control. 2022 Jun 13;11:82. doi: 10.1186/s13756-022-01123-w (PMC9195457; doi:10.1186/s13756-022-01123-w)
Supplement: Supplementary file 3 — Additional file 3: Table S2. Rate of MDRO Acquisition per 1000 Bed Days by organism, July 2014 – October 2020. [file 13756_2022_1123_MOESM3_ESM.docx]

**Supplementary Table 2.** Rate of MDRO Acquisition per 1000 Bed Days, July 2014 – October 2020

| **Organism** | **Events** | **Rate (95% CI)** |
| --- | --- | --- |
| MRSA | 67 | 2.9 (2.2-3.7) |
| VRE | 31 | 1.3 (0.9-1.9) |
| *Pseudomonas* (Group 1) | 81 | 3.5 (2.8-4.3) |
| *Pseudomonas* (Group 2) | 21 | 0.9 (0.6-1.4) |
| *Acinetobacter* | 15 | 0.6 (0.4-1.1) |
| *Stenotrophomonas* | 65 | 2.8 (2.2-3.6) |
| CRE | 11 | 0.5 (0.2-0.9) |
| ESBL-PE | 39 | 1.7 (1.2-2.3) |
| Total number of bed days: 23135.  CI = confidence interval; CRE = Carbapenem-resistant Enterobacteriaceae; ESBL-PE = Extended spectrum beta lactamase producing Enterobacteriaceae; MDRO = multi-drug resistant organism; MRSA = Methicillin-resistant *Staphylococcus aureus*; VRE = Vancomycin-resistant Enterococcus. *Pseudomonas aeruginosa* groups are defined by resistance to carbapenems (Group 1) or either piperacillin-tazobactam or cefepime (Group 2). | | |
