# Supplementary material for: Patterns of multidrug resistant organism acquisition in an adult specialist burns service: a retrospective review
Source: Antimicrob Resist Infect Control. 2022 Jun 13;11:82. doi: 10.1186/s13756-022-01123-w (PMC9195457; doi:10.1186/s13756-022-01123-w)
Supplement: Supplementary file 4 — Additional file 4: Table S3. Time to First MDRO Isolation since Admission. [file 13756_2022_1123_MOESM4_ESM.docx]

**Supplementary Table 3.** Time to First MDRO Isolation since Admission

| **Organism** | **N** | **Time to First MDRO Isolation** | **Isolated within 48 hours** |
| --- | --- | --- | --- |
| Any MDRO | 226 | 10.9 (5.6-20.5) | 29 (12.8%) |
| MRSA | 66 | 9.8 (2.7-24.2) | 15 (22.7%) |
| VRE | 31 | 14.9 (8.5-30.6) | 0 |
| *Pseudomonas* (Group 1) | 81 | 23.6 (15.7-36.0) | 0 |
| *Pseudomonas* (Group 2) | 21 | 20.1 (15.6-45.4) | 0 |
| *Acinetobacter* | 15 | 18.8 (12.1-24.2) | 0 |
| *Stenotrophomonas* | 65 | 9.3 (3.6-22.7) | 10 (15.4%) |
| CRE | 11 | 24.7 (10.3-40.4) | < 5 |
| ESBL | 39 | 9.8 (7.2-23.1) | < 5 |
| Time to swab data presented as median (IQR) days. Isolated within 48 hours data presented as frequency (percentage). Excludes missing data.  CRE = Carbapenem-resistant Enterobacteriaceae; ESBL-PE = Extended spectrum beta lactamase producing Enterobacteriaceae; IQR = interquartile range; MDRO = multi-drug resistant organism; MRSA = Methicillin-resistant *Staphylococcus aureus*; VRE = Vancomycin-resistant Enterococcus. *Pseudomonas aeruginosa* groups are defined by resistance to carbapenems (Group 1) or either piperacillin-tazobactam or cefepime (Group 2).  Note that one patient could develop an MDRO from multiple organism groups. | | | |
